# Genetic mixing for population management: From genetic rescue to provenancing

**DOI:** 10.1111/eva.13154

**Published:** 2020-11-06

**Authors:** Ary A. Hoffmann, Adam D. Miller, Andrew R. Weeks

**Affiliations:** ^1^ School of BioSciences Bio21 Institute The University of Melbourne Parkville Vic. Australia; ^2^ School of Life and Environmental Sciences Centre for Integrative Ecology Deakin University Warrnambool Vic. Australia; ^3^ Deakin Genomics Centre Deakin University Geelong Vic. Australia; ^4^ cesar Pty Ltd Parkville Vic. Australia

**Keywords:** adaptation, conservation, genetic variation, population size, revegetation

## Abstract

Animal and plant species around the world are being challenged by the deleterious effects of inbreeding, loss of genetic diversity, and maladaptation due to widespread habitat destruction and rapid climate change. In many cases, interventions will likely be needed to safeguard populations and species and to maintain functioning ecosystems. Strategies aimed at initiating, reinstating, or enhancing patterns of gene flow via the deliberate movement of genotypes around the environment are generating growing interest with broad applications in conservation and environmental management. These diverse strategies go by various names ranging from genetic or evolutionary rescue to provenancing and genetic resurrection. Our aim here is to provide some clarification around terminology and to how these strategies are connected and linked to underlying genetic processes. We draw on case studies from the literature and outline mechanisms that underlie how the various strategies aim to increase species fitness and impact the wider community. We argue that understanding mechanisms leading to species decline and community impact is a key to successful implementation of these strategies. We emphasize the need to consider the nature of source and recipient populations, as well as associated risks and trade‐offs for the various strategies. This overview highlights where strategies are likely to have potential at population, species, and ecosystem scales, but also where they should probably not be attempted depending on the overall aims of the intervention. We advocate an approach where short‐ and long‐term strategies are integrated into a decision framework that also considers nongenetic aspects of management.

## 1 BACKGROUND

Many native animal and plant species have highly fragmented distributions as a result of widespread habitat destruction and the ongoing onslaught of invasive species (Frankham et al., 2019). As a result, remnant populations are often small and isolated, rendering them vulnerable to the negative effects of genetic drift and inbreeding (Frankham, 2015; Weeks et al., 2011; Willi et al., 2013). These processes can lead to the expression of deleterious alleles (known as genetic load, see Willi et al., 2013) and reductions in overall population fitness (known as inbreeding depression, see Frankham, 1995), and elevate risks of maladaptation by compromising locally adapted traits (Lopez et al., 2009) and erosion of standing genetic diversity (Frankham, 2015; Weeks et al., 2011). The harmful effects of inbreeding depression and loss of genetic diversity are recognized as a major contributor to increasing global extirpation rates of small populations of sexually reproducing organisms (Markert et al., 2010; Ørsted et al., 2019; Speilman et al., 2004; Weeks et al., 2011). It has been estimated that gene flow is currently inadequate for overcoming these risks in 26% of invertebrate, 29% of vertebrate, and 55% of plant species persisting in fragmented landscapes (Frankham, 2015; Frankham et al., 2019).

The genetic integrity of many animal and plant populations is further compromised by rapid climate change which can decrease the adaptedness of local animal and plant populations (Aitken & Whitlock, 2013; Anderson & Wadgymar, 2020; Derry et al., 2019; Hoffmann & Sgrò, 2011). Standing genetic variation of small populations will be often insufficient for overcoming the requirements of keeping up with climate change (Aitken & Whitlock, 2013; Hoffmann & Sgrò, 2011; Willi et al., 2006). The fate of many populations will depend on the availability of genotypes that are pre‐adapted to future climates rather than de novo mutation (Carlson et al., 2014). These can be provided through gene flow between locally adapted populations distributed across environmental gradients (Kremer et al., 2012; Miller, et al., 2020). In the absence of gene flow (i.e., in fragmented landscapes), populations will become increasingly dependent on standing genetic variation within them and the generation of de novo mutations, both of which are greatly affected by small population size (Frankham et al., 2001, 2019; Weeks et al., 2011; Willi et al., 2006). In addition, inbreeding and genetic drift further exacerbate maladaptation in small populations (Weeks et al., 2011).

Although concerns about genetic integrity and future adaptation have largely focused on small populations, there is increasing interest in the vulnerability of large populations of functionally important species to extreme climatic events. These events can include the direct and large effects of fires, storms, and heat waves (Duke et al., 2017; Steel et al., 2019; Wernberg et al., 2010) as well as less direct effects such as changing species interactions following extreme events (Grant et al., 2017). The worlds’ coral reefs are a classic example of this vulnerability, with the frequency of bleaching events increasing dramatically over the last decade as sea water temperatures rise due to climate change (Hughes et al., 2018; van Oppen et al., 2017). Similarly, the recent Australian bushfire season in 2019–2020 resulted in over 97,000 km^2^ being burnt, major reductions in canopy and understory plant species, and the deaths of many millions of animals (Ward et al., 2020).

While large populations carry higher levels of genetic variance and novel mutations, the adaptive potential of large populations may nevertheless be insufficient for keeping pace with rapid climate change particularly for long‐lived species. Interventions may be needed to restore affected populations and safeguard remnant populations at risk of maladaptation (Quintero & Wiens, 2013). These challenges are prompting managers and practitioners to carefully consider the deliberate introduction of genotypes which are most likely to maximize the long‐term success and resilience of conservation and restoration programs, and ecosystem functionality under future environmental conditions. For instance, for forestry and the restoration of flora communities, this has led to shifts away from traditional local provenancing and toward mixed and targeted provenancing approaches that help to broaden the genetic basis of plantations, and include genotypes expected to be pre‐adapted to future environments (e.g., Breed et al., 2013; Williams & Dumroese, 2013).

As a result, there is a growing sense that direct genetic intervention may be needed to safeguard not only populations of threatened species, but also keystone species and the communities that rely on them in order to stall biodiversity loss and maintain ecosystem function. Strategies aimed at initiating, reinstating, or enhancing patterns of gene flow via the deliberate movement of genotypes around the environment are not new but are now gaining widespread interest from different quarters (Aitken & Whitlock, 2013; van Oppen et al., 2017; Ralls et al., 2018). Other strategies aimed at creating new genotypes that might be better adapted to current and future threats are also emerging, including the direct manipulation of allelic variants in threatened species (e.g., Samuel et al., 2020).

Our initial purpose here is to clearly define these strategies while pointing out the overlap and synergies between them and opportunities to combine their aims, but also to discuss potential conflicts. Our contention is that genetic mixing is at best viewed as a continuum, defined by the desired strategy specific outcomes, and also by the nature of the recipient and source population(s), elapsed time in achieving the outcome, and the associated risks involved. We start by providing some examples of the different genetic mixing strategies in Table 1. We then discuss them sequentially before coming back to integrate them based on their commonalities and differences. Note that this classification is not always consistent with the definition of terms by some other authors, with terms like genetic rescue being used more broadly in many instances, and we therefore restrict our use of these terms to definitions as given in Table 1.

**Table 1:** Classification of strategies related to genetic mixing

Type of mixing strategies	Definition based on underlying problem and strategy	Comments	Example
Genetic rescue	Deliberate genetic introductions aimed at masking of deleterious alleles responsible for genetic load in small populations leading to an increase in population growth rate	Term has also been used more broadly to encompass aspects of evolutionary rescue where there is an increase in fitness in a population following the introduction of new alleles (e.g., Whiteley et al., 2015). Probably, the most common example of genetic mixing considered so far but still relatively few practical applications	Bighorn sheep: marked improvements in reproduction, survival, and five fitness‐related traits. Trait values were increased by 23%–257% in maximally outbred individuals (Hogg et al., 2006) Mountain pygmy possum: hybrid fitness 2× higher than nonhybrids; larger body size, and female hybrids produced more pouch young and lived longer (Weeks et al., 2017)
Genotype provenancing	Introduction of genotypes into populations pre‐adapted to current or future conditions or (in the case of “mixed” provenancing) providing insurance against unpredictable conditions	Provenancing has a long history and was in the past mostly focused on identifying locally sourced genotypes for revegetation. The approach of sourcing of genotypes from different provenances to increase population adaptability (e.g., Broadhurst et al., 2008) is currently experimental but many trials in common gardens across gradients are being established particularly in tree species	*Pinus contorta* transplants across a latitudinal gradient to assess responses to thermal conditions at different times of the year (Montwé et al., 2018) Climate future plots of gray box (*Eucalyptus microcarpa*) and yellow box (*Eucalyptus melliodora*) where multiple provenances from regions climatically matched with future climates have been introduced into an area https://www.bushheritage.org.au/projects/nardoo‐climate‐ready‐revegetation
Evolutionary rescue	Reduction in extinction risk of populations facing environmental change due to adaptive evolution (e.g., Bell, 2017). The term refers to an increase and subsequent maintenance of adaptive genetic variability from assisted gene flow, or connecting population fragments into a metapopulation or increasing the size of an existing population to increase mutation and prevent the loss of genetic variability under drift	This process is supported by many theoretical models and experimental studies that show more rapid adaptation with larger population size, but applications are largely restricted to discussions of threatened species with fragmented distributions and low levels of genetic variation. Evolutionary rescue overlaps with genotype provenancing (which effectively leads to assisted gene flow in later generations)	Gene flow among isolated populations of Trinidadian guppies (*Poecilia reticulata*) that led to hybrids with increased fitness while maintaining characteristics associated with local adaptation (Fitzpatrick et al., 2020) Tests of adaptive variation in fragmented populations of dwarf birch (*Betula nana*) to investigate usefulness of gene flow to increase adaptive variation (Gentili et al., 2018)
Developing novel genotypes through species hybridization	Deliberate hybridization to generate novel genotypes that show heterosis and/or novel allele combinations with high fitness	Evidence usually relates to natural hybridization that has been tracked across time, but more recently deliberate experimental hybridizations are also being attempted	Interspecific hybridization followed by introgression has helped killifish (*Fundulus grandis*) adapt to pollution stress (Oziolor et al., 2019) Deliberate hybridization in *Acropora* corals leads to hybrids with high fitness and evidence from natural populations indicates high incidence of hybrid *Acropora* in marginal environments (Chan et al., 2018; Fogarty, 2012)
Developing novel genotypes through genomic selection	Identifying high fitness genotypes in populations for subsequent propagation	This technique is used extensively in agriculture but has not yet found much application in natural populations	Candidate genotypes of valley oaks (*Quercus lobate*) identified with faster growth under warmer temperatures have been identified (Browne et al., 2019)
Developing novel genotypes through allele modification	Direct manipulation of alleles through genetic techniques for subsequent release in natural populations	So far this has been proposed as an avenue for research, with some laboratory demonstrations, although no releases have been undertaken in natural populations	Generating birds with resistance to avian malaria where species are threatened (Samuel et al., 2020) Transgenic American chestnut trees resistant to fungal blight have been developed by inserting a gene from wheat, with the aim of restoring natural populations decimated by the pathogen (Newhouse et al., 2014)
Replacing species with other species having higher adaptive potential	Deliberate introduction of species with higher adaptive potential and/or reduced genetic load to increase population fitness and adaptability while maintaining functional processes in ecosystems	Not yet undertaken as far as we know but used in agriculture when cultivars are no longer suitable for an environment but a different crop with appropriate cultivars can be grown. Has also been considered in the context of heavily modified environments	The prestoration strategy as proposed for grasses (Butterfield et al., 2017) is linked to this idea Assisted evolution strategy for native plants in heavily modified environments (Jones & Monaco, 2009)

## 2 GENETIC RESCUE

Genetic rescue is the term given to the most widely known strategy for the deliberate movement of genes across populations. Most applications of this concept have been responsive in nature and directed at reducing the genetic load of small populations suffering from inbreeding depression and fitness declines as a consequence of close relatives mating and random genetic drift (Frankham, 2015; Tallmon et al., 2004; Whiteley et al., 2015). Genetic rescue has been the subject of several reviews recently (Bell et al., 2019; Whiteley et al., 2015); principally, the desired outcome of a genetic rescue is an increase in population fitness achieved through the masking of deleterious alleles that contribute to inbreeding depression. A key component for a successful genetic rescue is the mitigation of extrinsic threats so that the recipient population can grow, allowing selection to either purge deleterious alleles or decrease their frequency so that they are part of the populations segregating genetic load. A clear understanding of genetic load (Box 1) and the implications it has on the genetic rescue strategy is critical for selecting optimal source and recipient populations, and the longer‐term success of the genetic rescue.

**Genetic load**

Genetic load was originally defined by Haldane and Muller as the mutational load of a population reflecting the cumulative effects of mutations that accumulate in populations under mutation–selection balance (see Ewens, 2013). It now refers more broadly to the reduction in mean fitness of a population due to the accumulation of unfavorable alleles, relative to an idealized population composed of optimal genotypes (Whitlock & Davis, 2011). Genetic load is composed of drift load, mutation load, segregation load, recombination load, and migration load (Whitlock & Davis, 2011). Drift load and mutation load are important in conservation because they contribute significantly to the extinction risk of small populations and can impact on the success of different genetic intervention strategies, particularly genetic rescue.

*Mutation load* (also known as segregating load, not to be confused with segregation load) is the genetic burden of a population resulting from a steady flux of recessive deleterious mutations that remain at low frequency within populations. They are generated by mutation and natural selection acts to remove them, creating a low equilibrium frequency of deleterious alleles that reduces the overall fitness of the population (mutation–selection balance). When a population becomes small, inbreeding can lead to the elevation in frequency and expression of these segregating deleterious alleles, resulting in inbreeding depression (and likely further reductions in population size). If the population remains small and isolated, then some of the segregating load will be lost from the population and a fraction will become fixed due to random genetic drift. These alleles will be expressed throughout the population, contributing to the *drift load*, leading to reductions in population fitness and size, which feeds back to enhance *drift load*, which could result in extinction (Lynch et al., 1995). *Drift load* also represents the impact of drift when allele frequencies move away from their optimum values within a population.

Large populations are likely to carry a higher *mutational load* than small populations, whereas small populations will have a higher *drift load* than large populations (Lynch et al., 1995). However, populations that have been small for long periods of time are less likely to exhibit inbreeding depression than populations that have recently become small because they may have purged deleterious alleles over time. However, their *drift load* will continue to accumulate as mutations enter the population (known as mutational meltdown, see Lynch et al., 1995) and negatively impact population fitness and/or environmental resilience. Populations of a species can have different *mutation* and *drift* loads, and this is more pronounced when populations have been isolated for significant periods of time. This presents both an opportunity and a risk when considering mixing strategies such as genetic rescue and practitioners need to consider implications of combining individuals from populations with different genetic loads. For instance, moving individuals from a healthy large population into a small population suffering inbreeding depression (the classic genetic rescue scenario) is likely to result in the masking of deleterious alleles and a resulting increase in the fitness of F1 individuals. However, the genetic load of the recipient population may also eventually increase as new deleterious alleles become fixed. Population growth and selection can act to reduce the overall genetic load. However, if population growth does not occur, there could be fitness consequences of an increased genetic load after the genetic rescue. For instance, in the Isle Royale gray wolf, an initial positive effect of genetic rescue was later followed by a population decline (Hedrick et al., 2019).

Genetic rescue can also be applied as a preventative conservation tool to help overcome imminent risks of genetic load, inbreeding or lost genetic variation (adaptive potential). This may apply to populations recently fragmented (Bossuyt, 2007; Hogg et al., 2006), or small, isolated populations of long‐lived species yet to express noticeable fitness reductions due to generational lag (Miller et al., 2020). It can also apply to populations that are small and inbred, with a fixed genetic load, but not necessarily suffering inbreeding depression (Box 1). The influx of new genetic variants in these cases is akin to “genetic restoration” (Hedrick, 2005), but will also result in the masking of deleterious alleles that may be of immediate benefit to the population's fitness or may provide benefits under changing environmental conditions. Our definition of genetic rescue (which incorporates genetic restoration) differs somewhat to others as it is primarily focused on genetic load as the underlying mechanism, while we acknowledge that genetic rescue is also used in the context of plant self‐incompatibility systems (S allele variation; Willi et al., 2007). The benefits of genetic rescue have now been demonstrated in a variety of vertebrate, invertebrate, and plant species (Bell et al., 2019), and the potential for genetic rescue in conservation programs has recently been highlighted by Frankham (2015) but the approach has limitations if “rescued” populations remain small. This last point (rescued small populations need to be able to grow) has been overlooked in recent criticisms of genetic rescue (Robinson et al., 2020), as pointed out by Ralls et al. (2020), but is integral to the effective purging of deleterious alleles as discussed above (Box 2).

**A classic genetic rescue case study**

**Figure 1:**
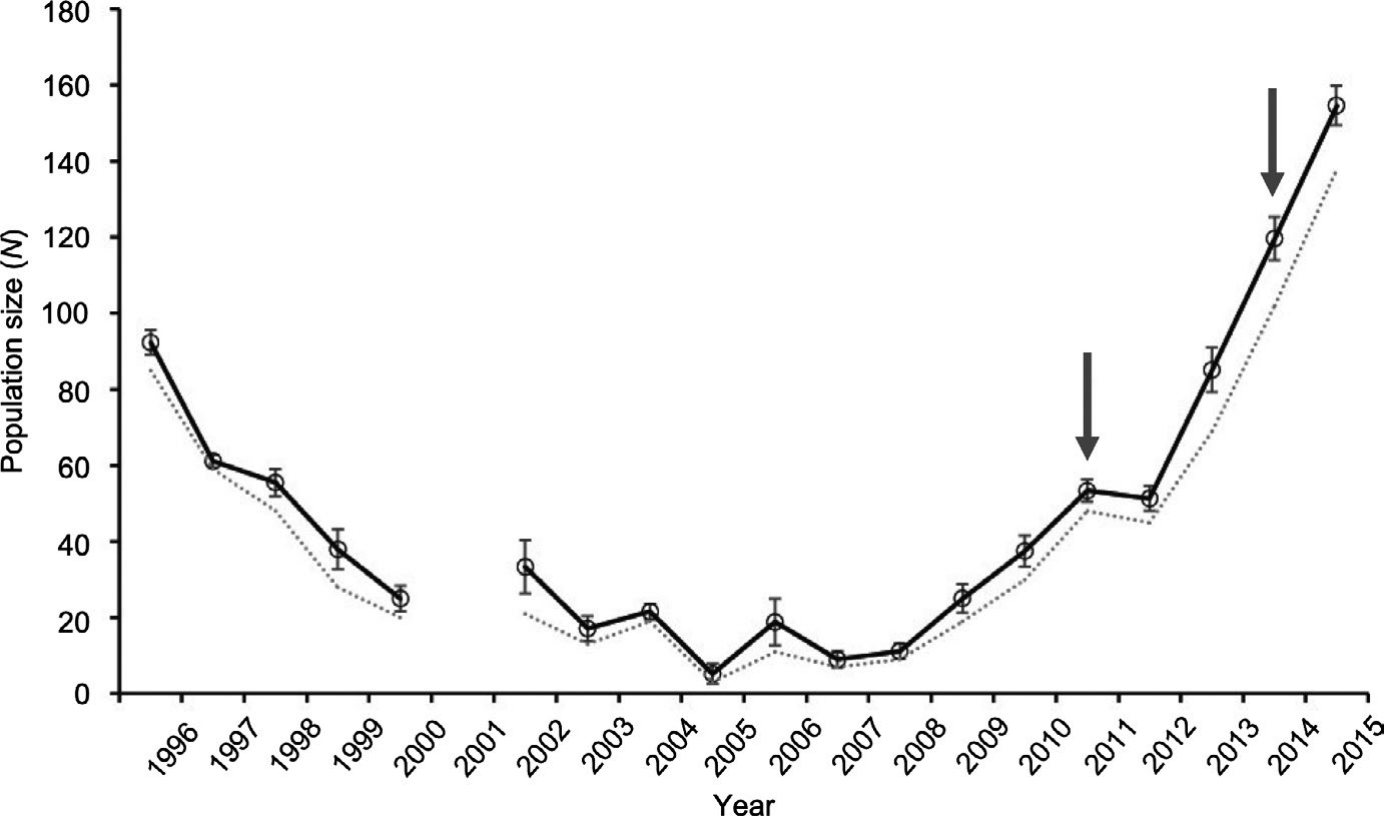
*Burramys parvus* adult population size at Mount Buller over time. Solid line is the population size estimate based on capture–recapture data with standard error (mean) bars. Dashed line represents the number of unique observed individuals. Arrows indicate the 2011 and 2014 introduction of six males from a central alpine region source population. Modified from Weeks et al. (2017)

*Burramys parvus* is one of Australia's most threatened marsupials and is restricted to alpine regions. The species persists in three main alpine regions, and these populations have been genetically isolated for at least 20,000 years (Figure 1). The southern population is restricted entirely within the Mount Buller alpine resort and suffered rapid demographic and genetic collapse in the late 1990s/early 2000s due to habitat degradation and fragmentation from resort activities (Mitrovski et al., 2008). Researchers assumed inbreeding depression through the fixation of deleterious alleles and a genetic rescue program was implemented along with habitat recovery and a predator control program (Weeks et al., 2017). A limited number of males from healthy and genetically variable *B. parvus* populations from the central region were introduced in 2011 and again in 2014. F1 and F2 hybrids were significantly fitter than nonhybrids; hybrid animals had a larger body size, and female hybrids produced more pouch young and lived longer. The population has shown rapid growth and is now at its highest level in 8 years following the initial genetic rescue. Continued growth is important for the population to reduce its genetic load (Box 1).

## 3 GENOTYPE PROVENANCING

The deliberate movement of genotypes across environmental gradients to help populations overcome risks of maladaptation is a common insurance genetic mixing strategy. The approach has been suggested within the context of managing long‐lived plants and safeguarding and restoring populations of keystone species threatened by environmental change. However, the approach is relevant to all long‐lived organisms where there is local adaptation across environmental gradients, but a lack of population connectivity across gradients. Single generation genotype mixing is particularly important where the maintenance of keystone species is critical for supporting other forms of biodiversity and ecosystem functionality.

Applications of genotype mixing as a tool for overcoming risks of maladaptation are becoming common in forestry and restoration plantings (Bucharova et al., 2019). Restoration practices have focused on local provenancing, which involves the use of local seed sources to reinforce existing plant populations or establish new populations where local can be defined through guidelines or genetic data (Krauss et al., 2013; McKay et al., 2005). These traditional practices were advocated based on the assumption that resident seed is best suited to local environmental conditions (O’Brien et al., 2007). However, this is being increasingly questioned due to apparent impacts of population fragmentation and rapid environmental change on the genetic integrity and adaptedness of local populations (Havens et al., 2015). Consequently, there has been a shift toward mixed/composite and targeted provenancing approaches in forestry and restoration plantings over the last decade (Broadhurst et al., 2008; Browne et al., 2019; Bucharova et al., 2019; Prober et al., 2015), with the intention of broadening the genetic basis of plantations, and providing at least some genotypes that enable adaptation to future environments (Sgrò et al., 2011).

This genetic mixing strategy typically applies to species with wide altitudinal or latitudinal ranges, many of which show genetically based clines across thermal and aridity gradients (Aitken & Bemmels, 2016; Halbritter et al., 2018; Jeffery et al., 2017; Pereira et al., 2017). A simple example of single generation genotype mixing might involve the movement of genotypes from current warm and dry‐adapted populations to colder and wetter locations. Ideally, the selection of genotypes to be mixed will be informed by experimental approaches and empirical data. Provenance trials provide an opportunity to test for adaptive genetic differentiation between local and distant populations by assessing the relative performance of genotypes under common garden conditions (field or controlled glasshouse environments; e.g., Montwé et al., 2018; Schmidtling, 1994; Thomson et al., 2009), while population genomic studies can help identify specific genotypes associated with phenotypes thought to be essential for countering stressful conditions (i.e., targeted gene rescue, see below [Browne et al., 2019; Holliday et al., 2017; Jordan et al., 2017; Sork et al., 2013]). In the absence of quantitative or correlative data, the selection of genotypes can be informed by climate profile matching approaches which help to identify source populations where current climates match those predicted for another part of the species distribution (Doherty et al., 2017; Jordan et al., 2017; Nitschke & Innes, 2008; Pina‐Martins et al., 2019).

The effects of climate change are spatially and temporally variable, yet many parts of the world are becoming warmer, drier, and more fire prone. While there might be opportunities to introduce genotypes that are pre‐adapted to aridity, fire, or thermal stress (Browne et al., 2019; Calvo et al., 2016), consideration needs to be given to the complex nature of climate‐induced selection pressures on species populations, which can be both direct and indirect and driven by interactions between biotic and abiotic factors. Consequently, we cannot be absolutely certain which genotypes will fare best under future environmental conditions. An insurance policy in the face of such uncertainty is best achieved by management that focuses on broadening the genetic basis of species populations, by supplementing local genotypes with a mix of genotypes from multiple populations (aligning with the principles of “composite provenancing”) in the hope that some of these will survive into the future (Broadhurst et al., 2008; Bucharova et al., 2019; Sgrò et al., 2011). While such approaches typically focus on single generation genetic mixing, recombination can produce novel gene combinations in subsequent generations, preserving locally adapted components of the genome. This leads to an overlap between provenancing and evolutionary rescue (see below) through re‐establishing connectedness (in the case of provenancing, connectedness may not be restored for some time beyond the initial influx of genetic material given that this strategy typically focuses on long‐lived species).

## 4 EVOLUTIONARY RESCUE

### 4.1 Evolutionary rescue through increasing population size and connectedness

Evolutionary rescue has been defined as adaptive evolutionary change that decreases the probability of extinction by restoring positive growth to a declining population (Carlson et al., 2014). The concept links to the notion that populations experiencing severe stress may avoid extinction through adaptation by natural selection. It differs from genetic rescue in that the population does not have to be small, inbred, or suffering from the expression of deleterious alleles (although genetic load and inbreeding can exacerbate the population decline). Similarly, it is different from provenance genotype mixing which targets an increase in variation across genotypes within one or two generations that are likely to be beneficial to a recipient population, although this strategy will also benefit longer‐term evolutionary changes. Evolutionary rescue can occur through standing genetic variation within a population or via the introduction of new genotypes through mutation and/or immigration (Bell, 2017). Evolutionary rescue lends from the principals of increasing genetic variation within a population to increase its adaptive capacity (Weeks et al., 2011) but will be slow in species with long generation times.

One of the core premises in evolutionary biology is that large populations are typically buffered from the effects of genetic drift and generally maintain high levels of genetic variation (accumulated via the process of mutation) which in turn increases their adaptive capacity (Hoffmann et al., 2017; Willi et al., 2006). This conjecture has been shown to apply in numerous laboratory experiments involving directional selection based on both traits and environments. In a recent example, adaptation across 10 generations in 120 *Drosophila* populations exposed to increasingly stressful laboratory medium was shown to correlate directly with variation in single nucleotide polymorphism markers across the genome but only weakly with inbreeding (Ørsted et al., 2019). Increasing population size with an associated increase in genetic variation should therefore promote evolutionary rescue. However, an increase in size by itself is likely to be insufficient when standing genetic variation is low—there must be an increase in genetic variation to help overcome risks of maladaptation. This will slowly occur in large populations through mutation, but the mutational increase in genetic variance is a slow process (Barrett & Schluter, 2008; Orr & Unckless, 2008) and unlikely to markedly increase the chance of evolutionary rescue occurring, particularly in rapidly changing environments.

An alternative is to initiate or re‐establish gene flow by connecting small isolated populations to increase effective population size and overall genetic variation (Box 3). Such approaches are expected to restore positive population growth and increase the potential for adaptation via evolution (Fitzpatrick et al., 2020) and have been promoted in threatened species metapopulation management (e.g., Pavlova et al., 2017). However, there is some risk of outbreeding depression due to the disruption of co‐adapted gene complexes (Edmunds, 2007; Frankham et al., 2011), the disruption of local adaptation due to the influx of maladapted genotypes (Fitzpatrick & Reid, 2019), and swamping of the native genome (Harrisson et al., 2019; Kim et al., 2018).

Population connections are expected to be particularly effective when gene flow is established among locally adapted populations spanning environmental gradients and where models indicate that they will enhance rates of adaptation (Kremer et al., 2012). Because of the importance of genetic variation in maintaining the adaptedness of populations, restoration efforts with plant species should attempt to capture as much genetic variation as possible compared to that present in natural populations, although this is not always successful (Jordan et al., 2019).

**Evolutionary rescue through connectedness case study**

**Figure 2:**
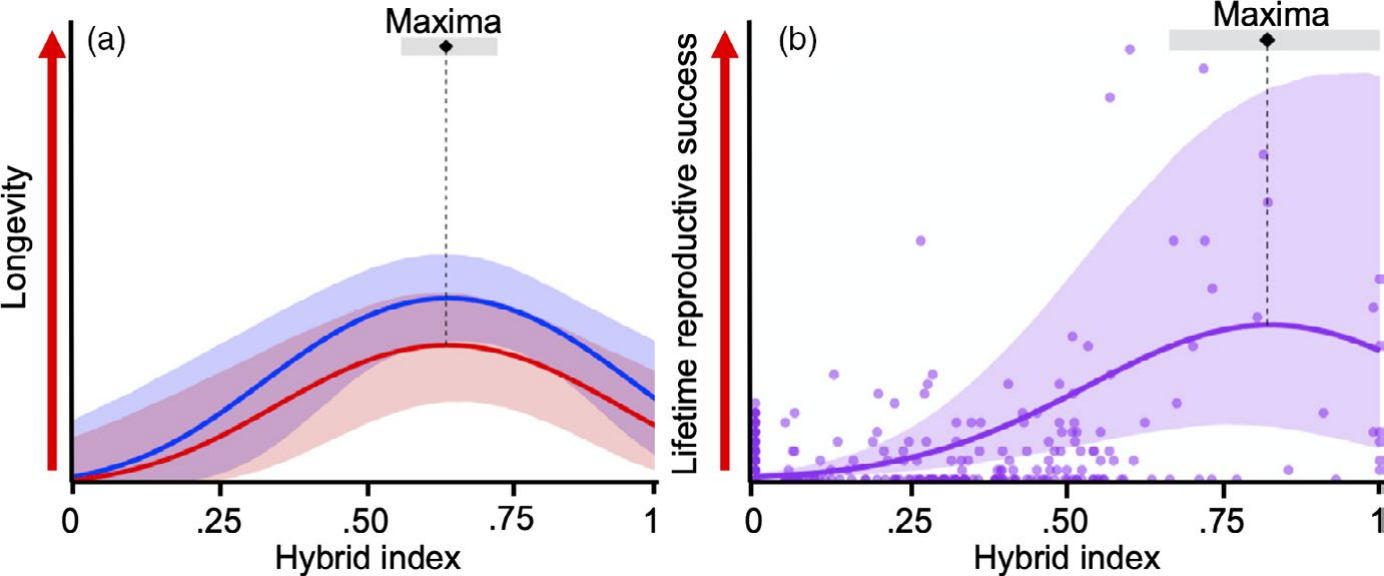
Fitness metrics (a) longevity and (b) lifetime reproductive success varied significantly with hybrid index (0, pure recipient genotype; 1, pure immigrant genotype). Blue and red lines indicate relative fitness between females and males, respectively. Shading around solid lines represents 95% confidence bands around regressions. Modified from Fitzpatrick et al. (2020)

Fitzpatrick et al. (2020) demonstrate intergenerational benefits of evolutionary rescue in wild fish populations. Controlled gene flow from a downstream population into small, inbred populations of wild Trinidadian guppies (*Poecilia reticulata*) with around 20× more genetic variation caused substantial increases in genomic variation, individual fitness, and population size. Fitzpatrick et al. (2020) reported 10‐fold increases in population size with hybrids living longer and reproducing more than residents and immigrants (Figure 2).

Multigenerational pedigrees indicated high hybrid fitness extending beyond heterosis in the F1 and up to six generations following gene flow. Despite substantial genomic changes and broadening of the genomic variation in recipient populations, genome scans indicate the maintenance of candidate adaptive allele frequencies following gene flow challenging traditional concerns for swamping effects.

### 4.2 Evolutionary rescue through novel genotype development

Novel genotypes can be created to increase the chance of evolutionary adaptation in populations where natural rates of adaptation through natural selection in populations with and without interventions are inadequate to deal with the rate and magnitude of environmental change. Here, we consider three types of novel genotype generation that have been proposed in the literature.

#### 4.2.1 Interspecific hybridization

Interspecific hybridization can result in the origin of novel genotypes by combining previously isolated gene pools and thereby exposing new gene combinations to natural selection. This process can rapidly accelerate rates of adaptation and has been recognized as an important process in evolutionary biology (Schwenk et al., 2008). However, the importance of interspecific hybridization in conservation biology remains contentious (Allendorf et al., 2001), particularly as unintended hybridization has occurred through anthropogenic activities and climate change may shift ecological niches that lead to new cases of hybridization, such as in polar and grizzly bears (Pongracz et al., 2017). There is, however, increasing interest in using interspecific hybridization to decrease extinction risks in natural populations (Hamilton & Miller, 2016). Recent research in corals highlights the benefits of interspecific hybridization for accelerating adaptation to a warming climate (Chan et al., 2018). Interspecific hybridization between rock wallabies in Australia appears to have occurred regularly throughout the recent and rapid radiation of the *Petrogale* species complex, and this is despite extensive interspecific chromosomal differences (Potter et al., 2015, 2017). Many plant species are also known to hybridize frequently in nature (Arnold, 1997), providing an ongoing source of novel genotypes for selection to act upon. Opposition to using hybridization in conservation generally comes from the species “purity” argument, the perceived risks of maladaptation and genetic incompatibilities (Muhlfeld et al., 2014), and the potential loss of the genome of the threatened species when the hybrid genome takes over (Harrisson et al., 2019; Harrisson et al., 2019). Conversely, hybridization is often raised as a way of preserving some of the genome for species on the brink of extinction (e.g., Garnett et al., 2011). Yet, the real value of interspecific hybridization should be considered long before this, as an opportunity to accelerate adaptive evolution and potentially prevent extinction or enhance the potential for evolutionary rescue (Box 4), which may require a restatement of conservation goals (Quilodrán et al., 2020). Any threat to local genomes could be tracked by tracking changes in genomes during hybridization as has been documented now in many cases (Todesco et al., 2016).

**Interspecific hybridization case study**

Oziolor et al. (2019) provide a case study of rapid adaptation and evolutionary rescue in killifish as a result of historical interspecific hybridization. Adaptive toxicant resistance has rapidly evolved in Gulf killifish (*Fundulus grandis*) that occupy habitats heavily polluted with halogenated and polycyclic aromatic hydrocarbons. Genome scans of *F. grandis* populations spanning a pollution gradient found that loci with the strongest signatures of recent selection harbor genes regulating aryl hydrocarbon receptor (AHR) signaling, which in turn is negatively correlated with pollution level. Comparisons of whole‐genome sequences from *F. grandis* and its sister taxon, the Atlantic killifish (*F. heteroclitus),* which has also adapted to similar chemical exposure, suggested that resistance loci in *F. grandis* introgressed within the last 30 generations from *F. heteroclitus*. The recent adaptive introgression was likely mediated by human‐assisted transport allowing for hybridization, but sufficiently rare to preclude extensive accumulation of deleterious genotypes in *F. grandis*.

#### 4.2.2 Marker‐based and genomic selection (harnessing existing genetic variation)

The process is akin to marker‐assisted selection seen in primary industries, which involves selecting for genetic variants linked to phenotypes of commercial interest. Primary industries have shifted their focus in the last decade to the use of marker‐based selection for enhancing both resilience and productivity of livestock, food crops, fisheries, and forestry under climate change. Genomic selection provides additional power in these analyses because a much higher density of genes is available for directing trait selection (Crossa et al., 2017; Isabel et al., 2020; Stranden et al., 2019; Yuan et al., 2019). Relevant examples include selection for heat resistance and decreased emissions in dairy cattle (Pryce & Haile‐Mariam, 2020) and a range of crop traits associated with climate change adaptation (Varshney et al., 2018).

There are few examples of genomic and marker‐based selection in wildlife conservation and restoration although are expected to increase as genomic sequencing costs decrease. Browne et al. (2019) recently used genomic approaches to identify candidate genotypes in Californian valley oaks (*Quercus lobata*) that promote fast growth under warmer temperatures and could be used to counter current negative effects of climate warming on growth rates (Box 5). Similarly, genotype–phenotype–environment association analyses have been used to identify specific genotypes involved in adaptation to cold hardiness in coastal Douglas fir (*Pseudotsuga menziesii var. menziesii*), which are at risk of frost damage under climate change (Vangestel et al., 2018).

Genotypes identified in such efforts could be used in provenancing or the introduction of new pre‐adapted genotypes into populations. However, there are challenges in using such an approach; for instance, there is an unpredictability in identifying useful genes and genotypes under natural environments. Quantitative genetic studies highlight that genotype–environment interactions tend to be substantial for traits in natural backgrounds (e.g., Huang et al., 2020; Monnahan & Kelly, 2017), meaning that the phenotypic effects of genotypes will depend on the environment in which an organism is reared. Thus, a favorable genotype from one environment may not be favored in a different environment. In contrast, levels of environmental variation in high yielding agricultural environments are typically low (Schou et al., 2020) and genotype–environment interactions are expected to be weaker given that environments are more homogeneous.

Good candidate markers for selection may emerge as genomic assessments of declining populations highlight genes and genomic regions implicated in climate change adaptation. For instance, in migratory yellow warblers (*Setophaga petechia*), populations with low frequencies of alleles associated with climate along gradients show the sharpest declines in population size (Bay et al., 2018). Such alleles could form the basis for future directed genetically based introductions. Several other studies on wild populations have pointed to genomic regions associated with climate that may be used to assess the vulnerability of populations (Ahrens et al., 2020; Jordan et al., 2017; Ruegg et al., 2018), although these studies are currently only correlative.

Importantly, stress responses in natural populations are often variable and likely to be highly polygenic, complicating our ability to identify specific alleles associated with particular phenotypic responses. Also, loci associated with traits in natural populations typically replicate poorly across populations (Schielzeth et al., 2018). Even selection experiments in replicate lines from the same *Drosophila* population for climate stress traits can produce quite diverse genetic outcomes with little overlap between replicate lines (Griffin et al., 2017). Even apparently simple traits like flowering time can have a complex genetic basis when considered across the distributional range of a species (Monnahan & Kelly, 2017; Zan & Carlborg, 2019). Only when traits are associated consistently with major genes is there much chance of detecting genotypes that are repeatedly associated with the same genetic changes. This holds in some cases of insecticide resistance evolution, such as the involvement of the voltage‐gated sodium channel gene in many cases of resistance to synthetic pyrethroids in different invertebrate species (Scott, 2019). Similarly, genes within the major histocompatibility complex (MHC) are often recognized as candidates for disease resistance in native wildlife (Ujvari & Belov, 2011). However, genetic strategies specifically focused on promoting MHC variability may fail if they do not adequately maintain genetic variation in other quantitative traits. There is an inherent danger that a focus on a few key genes such as the MHC locus may reduce standing genetic diversity across the genome, compromising the adaptability of populations to other stressors (Kardos & Shafer, 2018).

**Genomic selection case study**

**Figure 3:**
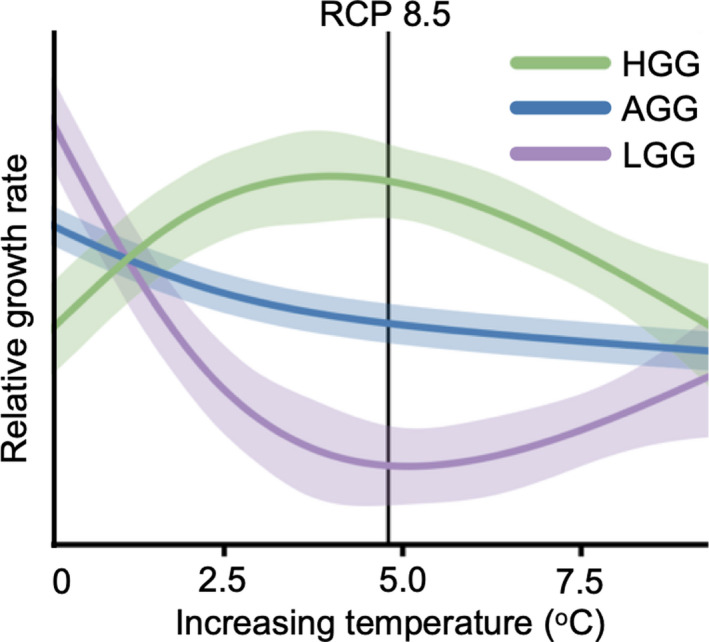
Relative growth rates of high, average, and low growth genotypes (HGG, AGG, LGG, respectively) at temperatures increasing from current day climates. Shading around solid lines reflects variation in growth around the mean growth profile. Modified from Browne et al. (2019)

Browne et al. (2019) show that an ecosystem‐foundational species in California, valley oak (*Quercus lobata*), is maladapted to current temperature climates and growth rates are expected to slow as temperatures rise over the next century (Figure 3). By combining genome‐wide sequencing with individual oak growth trait measurements from a large‐scale common garden experiment, Browne et al. (2019) identified genotypes likely to promote fast growth under warmer temperatures. The authors discuss the benefits of selecting seed sources based on genomic‐estimated breeding values to help combat the negative consequences of future climate warming on growth rates in valley oak. Specifically, targeted gene rescue via genomic selection which optimally matches individuals to future climates based on genotype–phenotype–environment associations.

#### 4.2.3 Creation of novel alleles and genotypes

Genome‐editing technologies have been under development for decades and could assist wildlife conservation by providing opportunities to engineer new alleles and adaptive traits (Phelps et al., 2020; Piaggio et al., 2017; Redford et al., 2014). Specifically, the clustered regularly interspaced short palindromic repeats (CRISPR) system offers the ability to target functional proteins to relatively precise locations in the genome and make modifications that change the coding sequence or activity of genes. While CRISPR technology is still at a starting point and major investments are outside of the conservation area, use of this technology to create gene drives that can rapidly spread beneficial genes through target populations may provide opportunities to rescue populations from risks of disease and climate stress in the future (Dearden et al., 2018). However, opportunities to engineer new adaptive traits will depend on the complexity of the trait being modified and face some of the same obstacles and challenges as genome‐based selection. For traits that increase disease resistance, CRISPR technologies may be applied to identify genetic alterations in related species or populations that have already acquired the necessary adaptation, perhaps with the potential future application of replicating those changes in target populations (Phelps et al., 2020; Piaggio et al., 2017). However, this is likely to be challenging for polygenic traits, and particularly if there are strong genotype–environment interactions which may result in the selection of alleles with a high fitness in one environment but a low fitness in different environments.

An issue for natural populations is that the genetic correlation for fitness‐related traits across environments can be weak and even negative (Sgrò & Hoffmann, 2004), reflecting the possibility that the relative fitness of genotypes can switch across environments and across time. We therefore suspect that genetic mixing strategies will tend to remain focused on the approaches discussed above unless there is trait variation with a relatively simple genetic basis and consistent phenotypic effects across environments aimed at specific problems such as pesticide resistance, which has been targeted by CRISPR approaches in agriculture (e.g., Sun et al., 2016). Instead, the initial applications of CRISPR in conservation will probably focus on monitoring variation at adaptive loci across the genome where current random sequencing approaches provide limited coverage (Phelps et al., 2020). De‐extinction (resurrecting extinct species) (Seddon et al., 2014) is also often raised as a possibility with CRISPR technology, but the above issues are only amplified to a much greater extent and currently impractical to implement.

## 5 FUNCTIONAL RESCUE

The above forms of genetic mixing apply not only to decreasing extinction risks but also maintaining ecosystem functions as populations of species with high functional importance continue to deteriorate. Management aimed at promoting the resilience of species that act as ecosystem engineers (i.e., keystone and foundation species) is particularly important, given their response to environmental change will have major impacts on overall community structure and ecosystem function. Many species providing critical ecosystem services (e.g., canopy‐forming tree species and marine macrophytes) are declining as a result of climate and other interacting stresses, leading to ecological cascade effects that compromise biodiversity and ecosystem function (e.g., Filbee‐Dexter & Wernberg, 2018; Stevens‐Rumann et al., 2018). Such declines also compromise the economic viability of primary industries such as forestry and wild harvest fisheries that are dependent on resilient target species and functional ecosystems.

Aside from directing genetic mixing efforts toward bolstering population resilience in functionally important species, another way of maintaining ecological functions is to replace keystone species that are failing to adapt to climate change and other stresses with species that can deal with the newly developed conditions. These species may already be present locally or in close proximity, but capable of surviving future environmental conditions and providing essential ecosystem services (“prestoration” Butterfield et al., 2017). Conversely, candidate species may not occur locally or be historically known from an area (“assisted migration” or “introduction”), but in that case there is the potential for species to have negative ecological impacts, particularly if introductions are into intact rather than disturbed communities (Peterson & Bode, 2020).

From a genetic perspective, an important component of species replacement relates to the ability of the replacement species to cope with the new conditions and undertake evolutionary rescue if a threat arises. To distinguish this aim from genetic and evolutionary rescue, we here use the term “functional rescue” (Table 1). This application is enmeshed with notions around provenancing as well as evolutionary rescue. It represents a way in which the decline in keystone species might be reduced via the expansion of species with a higher intrinsic ability to cope with future stressful conditions (e.g., grasses dealing with drier conditions [Butterfield et al., 2017]) or adapt to those conditions once they develop (e.g., widespread species with a higher intrinsic adaptive capacity than narrowly distributed species, as illustrated in comparisons of species of flies [Bush et al., 2016] but likely to apply much more generally [Diamond, 2018]). Functional rescue, while relatively controversial, may play an increasingly important role in balancing ecosystems if keystone species continue to be placed under increasing pressure due to rapid environmental change.

## 6 SOURCE AND RECIPIENT POPULATIONS

When considering strategies covered above, their success will depend on the source population on which the strategy is based, the time frame involved, the ecological context (which impacts on whether populations can expand or are destined to remain small), and the history of the recipient population (Ralls et al., 2020; Weeks et al., 2011). In Table 2, we consider three types of recipient populations in which a rescue might be attempted that vary in their history (we ignore genetic provenancing here but acknowledge that this can occur into a range of populations to produce immediate fitness benefits related to the novel alleles that are introduced). The first consists of a population that has been small for some time which might occur on a conservation reserve, an island, or some other fragment from which it cannot easily expand. This is the type of situation where a population has been threatened for many generations. Such populations may have purged deleterious alleles over time, but nonetheless still carry a fixed load that has reduced fitness, and the population is also likely to have an overall low level of genetic diversity. The second situation is similar to this, but the reduction in population size is relatively recent, resulting in a population becoming recently threatened. In this case, any purging of mutational load (segregating deleterious alleles) has not yet occurred, but some genetic variation may have been lost by drift and inbreeding depression effects may be substantial due to deleterious alleles becoming fixed or occurring in high frequency. In the third category, a population that has been small for some time is now able to expand, where ecological restoration has been successful at providing vacant ecological space for the species (such as the Mountain Pygmy possum example in Box 2).

**Table 2:** Likely benefits (+) and costs (−) of different types of genetic mixing strategies

Genetic mixing strategy	Source population size	History of recipient population size		
		Persistently small	Small, recent decline	Small in past but potential to expand^a^
Immediate effects (F1, F2)				
Genetic rescue (overcoming genetic load by masking deleterious alleles)	Small	+ → ++	+ → ++^b^	+ → +++
	Large	+ → ++	+ → ++^b^	+ → +++
Evolutionary rescue (through increasing connectedness)	Small^c^	+	+	+
	Large	++	+	++
Longer‐term effects (F3+)				
Genetic rescue (continued accumulation of deleterious alleles)	Small	‐‐	‐‐	=^d^
	Large	‐‐‐	‐‐‐	=^d^
Evolutionary rescue (through increasing connectedness)	Small	=	=	+
	Large	+	+	+++

Benefits can be immediate (F1, F2 stage) or longer term (F3+) and will depend on the source population(s) available for genetic material and the population size history of the recipient population. = represents same effect as in immediate effects. The advantages and costs are separated into those associated with genetic versus evolutionary rescue as defined in Table 1.

^a^ Assumes population continues to expand as extrinsic threats are mitigated.

^b^ Depends on level of inbreeding during decline.

^c^ Depends on some beneficial alleles being introduced from the small source population.

^d^ Assumes effective purging of deleterious alleles (decrease in genetic load).

The effect of a genetic rescue on these three populations will depend on the nature of the source populations providing genetic material for the rescue, which are expressed as two extremes (small or large population) in Table 2. Regardless of the source population size, the immediate effects of a genetic rescue on a small recipient population should involve masking of deleterious alleles (mutation and drift loads), particularly if there is a recent history of decrease in population size and the segregating (mutation) load is increasing in frequency within the population (Table 2a). There is increasing evidence of such recovery (Frankham, 2015; Ralls et al., 2020), which may be substantial if the population is already suffering from inbreeding depression. Recent evidence (Harrisson et al., 2019; Harrisson et al., 2019; Huisman et al., 2016) suggests that even small increases in inbreeding can have substantial effects on the lifetime fitness of small natural populations, highlighting the potentially large positive effect of this type of rescue. On the other hand, the genetic load of a population that has recently decreased in size may still be mostly segregating at a lower frequency and therefore masking has less effect, emphasizing the importance of considering past evolutionary history. Unfortunately, it is difficult to collect data on the impact of purging in populations of threatened species but experiments on model organisms point to its effectiveness as long as inbreeding is slow (Hedrick & Garcia‐Dorado, 2016). Over the longer term, there is a risk that genetic rescue aimed at masking deleterious alleles can have perverse effects when recipient populations remain small (Hedrick et al., 2019). New deleterious alleles introduced into small populations may then contribute to inbreeding depression as an increasing number of matings occur among related individuals. This is a significant risk when a recipient small population is unable to grow or grows too slowly (due to a lack of habitat or other constraints) and could lead to an even more perverse outcome (Box 1) unless alleles that are beneficial are being introduced; such risks need to be carefully managed with the nature of source populations likely to be important (Ralls et al., 2020). Ideally, ecological restoration and mitigation of extrinsic threats allowing recipient populations to expand should therefore accompany genetic rescue attempts (Weeks et al., 2017).

In terms of provenancing and genome selection, deliberate introductions of pre‐adapted genotypes can help overcome risks of maladaptation if the selection process can capture the challenges involved in defining the current and future fitness of the selected genotypes (Montwé et al., 2018). In contrast, the immediate benefits of evolutionary rescue through re‐establishing connectedness are less clear and will depend on the nature of the source populations (Weeks et al., 2011) and a myriad of other factors, such as trait heritability, genetic interactions among traits, and environmental effects including epigenetic effects. If source populations are small and do not contain genotypes adapted to existing or emerging conditions, there will be few benefits for enhancing rates of evolution in the recipient population. On the other hand, re‐establishing gene flow with large source populations should increase rates of subsequent evolutionary change as the effective size of a population is increased, as illustrated experimentally (Ørsted et al., 2019). This will be particularly the case if the recipient population is expanding following ecological restoration and/or the development of corridors that facilitate gene flow.

Finally, the risk of genetic incompatibility between source and recipient populations should be mentioned. This is likely to be greater if source and recipient populations are further separated in evolutionary time when the extent of local adaptation through co‐adapted loci is increased, particularly if there have been chromosomal rearrangements that distinguish populations (Frankham et al., 2011). This issue is often discussed but rarely evaluated and crosses between populations separated by many thousands of years and generations can produce evidence of heterosis with little outbreeding depression (Wells et al., 2019).

## 7 CONSIDERING MULTIPLE OPTIONS

Genetic rescue and evolutionary rescue will interact to dictate the final impacts on populations. The term “rescue” typically does not relate to a specific timeframe, although measurable outcomes in natural populations are typically short term (Fitzpatrick et al., 2020; Weeks et al., 2017). What might be considered as “rescued” at one point in time might not be so at a later point (or vice versa), depending on the genetic mechanism that predominates. An immediate benefit of genetic rescue might happen after a single generation with F1 individuals displaying hybrid vigor through the masking of deleterious alleles; however, further accumulation of deleterious genes through inbreeding following rescue may manifest in subsequent generations. Outbreeding depression represents another risk as segregation of alleles occurs (breakup of locally adapted alleles, expression of deleterious alleles, etc.), although this might be a transient state in larger “rescued” populations where ongoing selection will likely remove any deleterious allele combinations.

**Figure 4:**
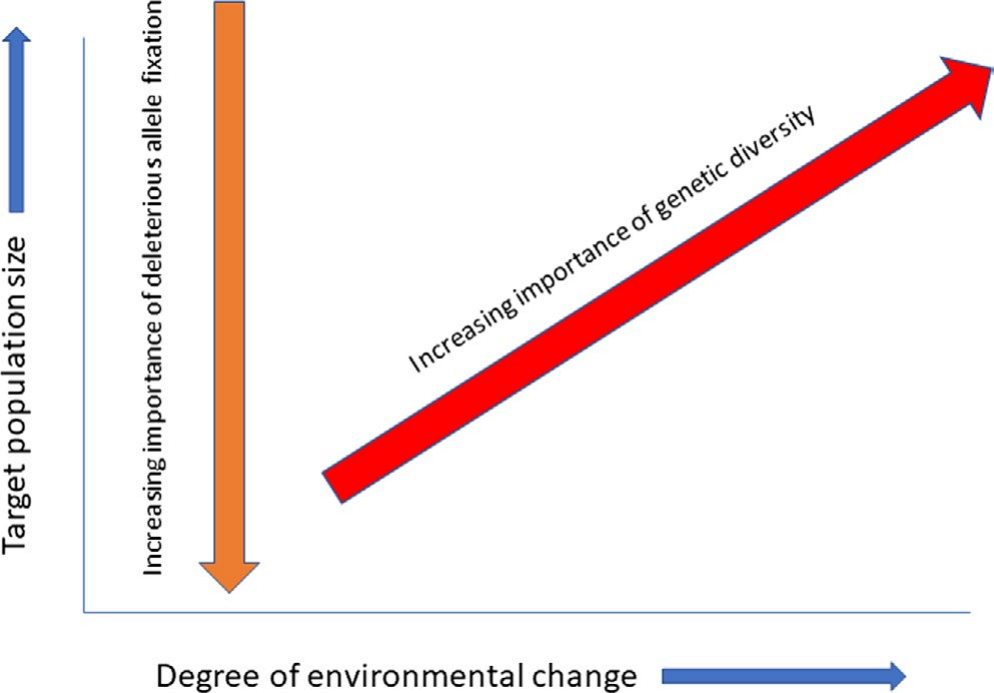
Population size versus environmental change in genetic mixing strategies. As recipient population size decreases, the potential impact of deleterious alleles reaching fixation increases, and the focus shifts to genetic rescue. However, as rates of environmental change experienced by the recipient population increase, the focus shifts to adaptive genetic diversity

The various components contributing to rescue and provenancing may establish a conflicting scenario between various outcomes of an intervention, depending on the timescale, recipient population, and environmental variability (Figure 4). In large populations exposed to substantial environmental changes, the success of a rescue will be very much based on generating adaptive diversity. On the other hand, the rescue of small populations will depend on the effective purging of deleterious alleles, although with rapid environmental change such populations may also still need to adapt to persist. Moreover, the purging process is complicated by the fact that the deleterious effects of many mutations contributing to inbreeding are environment dependent (Bijlsma et al., 1999; Cheptou & Donohue, 2011). Consequently, rescue attempts must focus not only on masking deleterious alleles, but also enhancing levels of genetic diversity, while ensuring population growth that helps to maintain genetic diversity. Populations that are forever destined to be small (i.e., where habitat constraints limit potential for substantial population growth) may therefore require ongoing management to ensure the purging of deleterious genes, and the maintenance of genetic diversity. Connecting these populations through the ongoing deliberate movement of individuals will be critical for their effective management.

The challenge in devising appropriate strategies for a specific situation is to consider the different factors that could contribute to population persistence. This requires a level of information that is unlikely to be available in most situations. For instance, how much inbreeding depression occurs in a population? In some situations, it may be substantial (e.g., Harrisson et al., 2019; Harrisson et al., 2019), but in others, populations may persist regardless of inbreeding, with very little depression in fitness taking place (e.g., Carleial et al., 2017). If inbreeding depression is currently minimal, it might yet develop once the environment changes, particularly if it becomes more stressful. Similarly, inbreeding effects may simply not have had time to manifest, particularly when population declines have been relatively recent. The data on inbreeding depression and stress are quite mixed (Armbruster & Reed, 2005); in some situations, stress might have little impact, unless novel conditions are encountered (Carleial et al., 2017), yet in others it is greatly enhanced under stress (Dahlgaard & Hoffmann, 2000; Fox & Reed, 2011). Similarly, how much evolutionary rescue is possible in a population? As already discussed, this will depend on the nature of the source and recipient populations, and preliminary data on adaptive potential coming from genomic studies, common garden experiments, and translocations. If local adaptation is absent due to epigenetically modified alleles rather than DNA‐encoded differences, it may be that there is little to be gained from the introduction of new genotypes into populations from conditions that match those predicted for the future. On the other hand, differentiation among populations that is genomically encoded and linked to the future conditions of a recipient population will likely lead to substantial benefits of an evolutionary rescue.

## 8 GUIDELINES AND DECISION FRAMEWORKS

A number of guidelines have been developed for the implementation of genetic mixing strategies. These include guidelines aimed at deciding whether genetic rescue should be implemented (e.g., Ralls et al., 2018) and guidelines around provenancing in revegetation (Breed et al., 2018). In addition, there is a detailed set of practical guidelines in Frankham et al. (2019) that provides relevant material on decision making, particularly within the context of genetic rescue. The set of chapters in these guidelines provides information that can assist in computing parameters that help in the assessment of relatedness among populations, levels of gene flow, estimating inbreeding levels, etc., and it also highlights areas where information is often lacking.

**Figure 5:**
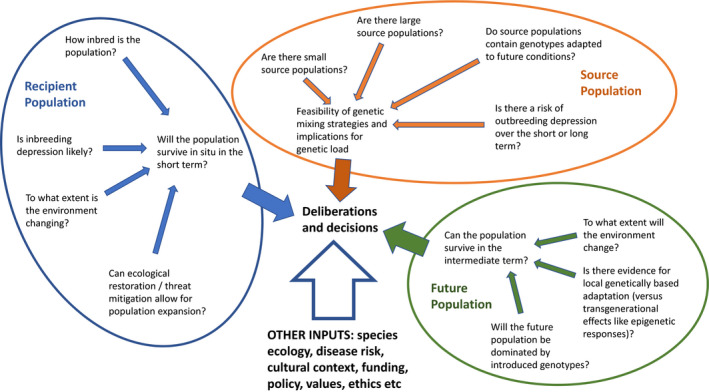
A framework for making management decisions around the implementation of genetic mixing. All components of the framework need to be considered in determining investment even if data are imperfect

Our approach here is to start off with the premise that there will be many unknowns when making decisions around genetic mixing but that decisions will nevertheless need to be made. We have summarized the various factors that need to be weighed up in Figure 5. These highlight inputs required to assess the immediate threats to a population posed by inbreeding, the longer‐term threats associated with environmental change that could result in populations unable to persist without adaptation, and the opportunities provided by genetic mixing in alleviating these threats. By presenting the options in this way, we hope to promote discussion within expert groups involved in providing management advice around the broader context of genetic mixing within local contexts. While there are many situations where genetic mixing could be applied (Ralls et al., 2018), we favor a cautionary approach where all possible complications and opportunities are identified and where an appropriate timeframe is considered which encompasses the future challenges posed by climate change and habitat loss.

In acknowledging the uncertainty around many of the variables that need to be considered, it is critical to undertake ongoing monitoring (Frankham et al., 2019). As best practice, we do not advocate a reliance on “rules” because parameters can vary so much among populations and species. For instance, the impacts of genetic rescue may vary depending on the populations crossed as in the case of the shrub *Grevillea repens* (Holmes et al., 2008). There is little correlation between genetic distance and the extent of rescue benefits, preventing this simple metric from being used (Holmes et al., 2008; Willi et al., 2007). Decisions based on immediate benefits may be appropriate, such as where two small populations are combined in cases where a population may otherwise decline rapidly such as in the case of robins in New Zealand (Heber et al., 2013) and wolves in Europe (Åkesson et al., 2016), but the long‐term danger and detrimental effects of accumulating deleterious alleles in small populations should be recognized. Immediate benefits are also important in long‐lived species from rapidly changing environments where local provenances are not expected to persist (Sgrò et al., 2011). Adaptation to rapid environmental change needs to be increasingly considered given that environmental change is already resulting in a high rate of species turnover (e.g., Lewthwaite et al., 2017; MacLean et al., 2018). Adoption of any genetic mixing strategies will need to work together with other aspects of conservation and restoration programs regardless of whether the focus is on threatened species or on maintaining keystone species in the environment. This includes population structures that minimize the risk of catastrophic events leading to extinctions as well as considering disease risks and cultural/policy perspectives (Figure 5). Within the context of future climate change, there are many nongenetic factors to consider in any vulnerability assessment (Foden et al., 2019).

## 9 CONCLUSIONS

Genetic mixing (and rescue) strategies aimed at deliberate interventions in populations cover a continuum depending on genetic and evolutionary goals and the timeframes involved. When modifying populations to ensure some sort of rescue outcome, it is critical that strategies take into consideration the scenarios and endpoints of a rescue being considered (Figure 2). The scenarios depend on (a) history of population size in the recipient population, (b) potential for population expansion, (c) immediate and future environmental threat level, and (d) availability of other populations as sources for genetic augmentation, including their size, location, and genetic basis. The potential for outbreeding depression is an important consideration where genetic mixing is proposed between evolutionary distant source populations (or interspecific hybridization); however, this does not necessarily impact overall success, particularly if the state is transitory and a population can purge deleterious mutations.

Appropriate scenarios for deciding on genetic mixing strategies also depend on the nature of source populations available for mixing. Where these are all small, resilience should be built up by sourcing from multiple small populations, acknowledging that population size needs to increase to avoid perverse outcomes of a higher genetic load. However, when masking inbreeding depression, sourcing from large populations produces additional issues because these populations have not been purged in the same way as persistently small populations. If only a single large source population is available and inbreeding continues in the recipient population, then this could lead to further inbreeding depression due to an inability to purge the genetic load, whereas a continuous influx from a large population would prevent this issue occurring.

Applications of genetic mixing will also depend on the nature of the species being considered and particularly their generation time within the context of the rate of environmental change. While inbreeding depression masking may be most relevant to F1s, there are many situations where such a short time frame is critical such as in wildlife with long generation times. The different approaches carry different levels of risk. So evolutionary rescue through assisted gene flow is recommended for large populations with clear evidence of adaptation likely to benefit most from this process (Aitken & Whitlock, 2013). Decisions around the right strategy will depend on many factors and priorities in individual cases, and ultimately, the definition of success.

## CONFLICT OF INTEREST

None declared.

## Data Availability

No data are used in this paper.
